# Practical considerations for navigating the regulatory landscape of non-clinical studies for clinical translation of radiopharmaceuticals

**DOI:** 10.1186/s41181-022-00168-x

**Published:** 2022-07-19

**Authors:** Aruna Korde, Renata Mikolajczak, Petra Kolenc, Penelope Bouziotis, Hadis Westin, Mette Lauritzen, Michel Koole, Matthias Manfred Herth, Manuel Bardiès, Andre F. Martins, Antonio Paulo, Serge K. Lyashchenko, Sergio Todde, Sangram Nag, Efthimis Lamprou, Antero Abrunhosa, Francesco Giammarile, Clemens Decristoforo

**Affiliations:** 1 Department of Nuclear Sciences and Applications, International Atomic Energy Agency (IAEA), Vienna International Centre, PO Box 100, 1400 Vienna, Austria; 2 Radioisotope Centre POLATOM, National Centre for Nuclear Research, Andrzej Soltan 7, 05-400, Otwock, Poland; 3 Department of Nuclear Medicine, University Medical Centre Ljubljana, 1000 Ljubljana, Slovenia; 4 Faculty of Pharmacy, University of Ljubljana, 1000 Ljubljana, Slovenia; 5 National Centre for Scientific Research “Demokritos”, Institute of Nuclear & Radiological Sciences and Technology, Energy & Safety, 15341 Athens, Greece; 6 Department of Immunology, Genetics and Pathology, Ridgeview Instruments AB, Uppsala Universitet, Dag Hammarskjölds Väg 36A, 752 37 Uppsala, Sweden; 7 Bruker BioSpin MRI GmbH, Rudolf-Plank-Str. 23, 76275 Ettlingen, Germany; 8 Nuclear Medicine and Molecular Imaging, Katholieke Universiteit Leuven, 3000 Louvain, Belgium; 9 Department of Drug Design and Pharmacology, Faculty of Health and Medical Sciences, University of Copenhagen, Jagtvej 160, 2100 Copenhagen, Denmark; 10 Department of Clinical Physiology, Nuclear Medicine and PET, Copenhagen University Hospital, Blegdamsvej 3, 2200 Copenhagen, Denmark; 11 Institut de Recherche en Cancérologie de Montpellier (IRCM), INSERM U1194, Institut Régional du Cancer de Montpellier (ICM), Université de Montpellier, 34298 Montpellier, France; 12 Department of Preclinical Imaging and Radiopharmacy, Werner Siemens Imaging Center, Eberhard Karls University Tübingen, Röntgenweg 13/1, 72076 Tübingen, Germany; 13 Cluster of Excellence iFIT (EXC 2180) “Image-Guided and Functionally Instructed Tumor Therapies”, University of Tübingen, Tübingen, Germany; 14 Centro de Ciências E Tecnologias Nucleares, Instituto Superior Técnico, Universidade de Lisboa, Bobadela Lrs, Campus Tecnológico e Nuclear, Estrada Nacional 10, Km 139.7, 2695-066 Lisbon, Portugal; 15 Department of Radiology, Memorial Sloan Kettering Cancer Center, New York, NY USA; 16 Department of Medicine and Surgery, University of Milano-Bicocca, Tecnomed Foundation, Milan, Italy; 17 Department of Clinical Neuroscience, Centre for Psychiatry Research, Karolinska Institutet and Stockholm County Council, 171 76 Stockholm, Sweden; 18 Bioemtech, Lefkippos Attica Technology Park-N.C.S.R Demokritos, Athens, Greece; 19 ICNAS/CIBIT, Institute for Nuclear Sciences Applied to Health, University of Coimbra, Coimbra, Portugal; 20 Department of Nuclear Medicine, Medical University Innsbruck, 6020 Innsbruck, Austria

**Keywords:** Radiopharmaceuticals, Regulations, Non-clinical testing, IAEA, Clinical translation, Preclinical development

## Abstract

**Background:**

The development of radiopharmaceuticals requires extensive evaluation before they can be applied in a diagnostic or therapeutic setting in Nuclear Medicine. Chemical, radiochemical, and pharmaceutical parameters must be established and verified to ensure the quality of these novel products.

**Main body:**

To provide supportive evidence for the expected human in vivo behaviour, particularly related to safety and efficacy, additional tests, often referred to as “non-clinical” or “preclinical” are mandatory. This document is an outcome of a Technical Meeting of the International Atomic Energy Agency. It summarises the considerations necessary for non-clinical studies to accommodate the regulatory requirements for clinical translation of radiopharmaceuticals. These considerations include non-clinical pharmacology, radiation exposure and effects, toxicological studies, pharmacokinetic modelling, and imaging studies. Additionally, standardisation of different specific clinical applications is discussed.

**Conclusion:**

This document is intended as a guide for radiopharmaceutical scientists, Nuclear Medicine specialists, and regulatory professionals to bring innovative diagnostic and therapeutic radiopharmaceuticals into the clinical evaluation process in a safe and effective way.

## Background

Radiopharmaceuticals (RPs) fall into the general category of drugs or Medicinal Products as defined in current legislation in the US and Europe (Directive 2001; Practice and for Positron Emission Tomography Drugs 2022) and several pharmacopoeia monographs. Hence, they are subjected to pharmaceutical, health, and radiation safety considerations. The current heterogeneous regulations among different countries are detrimental to the growth of the dynamic field of RPs. The International Atomic Energy Agency (IAEA) has been addressing this issue through various activities. A recent Technical Meeting conducted by the IAEA on `Preclinical testing of radiopharmaceuticals’ provided the opportunity for the participating experts to discuss experiences related to preclinical testing and translational studies for RPs. They identified the major challenges and requirements for guidance on this topic that might help RP researchers, professionals, and regulators. This document is not to be considered as a stand-alone guidance, rather, it provides specific features for non-clinical testing of RP which are essential key considerations from a regulatory perspective for clinical translation.

**Fig. 1:**
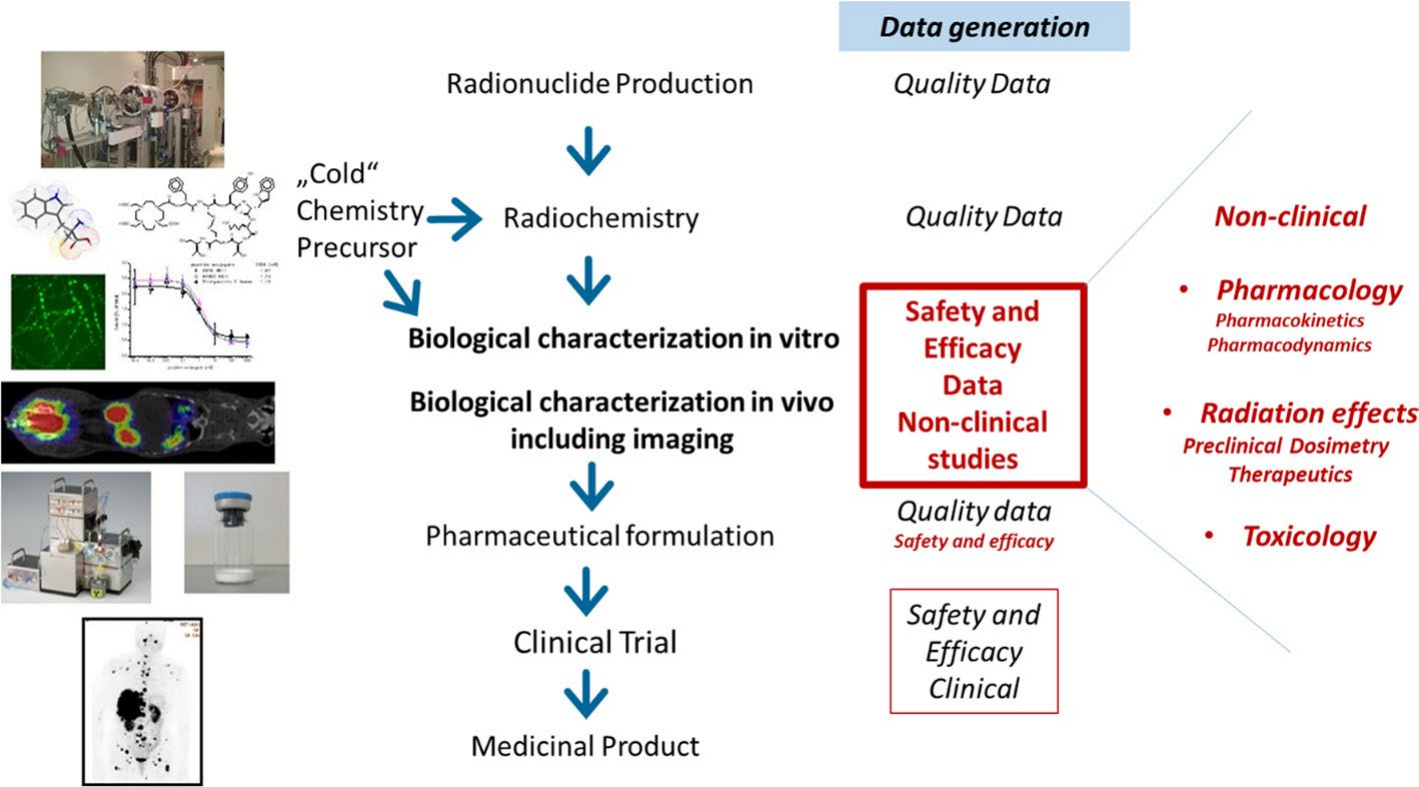
Clinical translation of radiopharmaceuticals: the development of a radiopharmaceutical requires many steps to finally be available for clinical use as a medicinal product, starting from the production of the radionuclide and precursor, thenradiochemical development to the biological characterization, pharmaceutical formulation and human studies within clinical trials. Data need to be generated regarding the chemical and pharmaceutical quality “Quality data”, but also data to predict safety and efficacy before human applications. These non-clinical data cover pharmacology, radiation effects and toxicology

The novel investigational use of RPs in human subjects carries inherent risks of eliciting unknown and unwanted effects. Therefore, besides providing detailed data on the chemical/pharmaceutical quality of a novel RP, prior to investigation in human subjects a series of experimental studies must be conducted to provide supportive evidence for the expected in vivo behaviour of the product of interest in humans (US FDA Guidance 2022; US FDA Guidance. Microdose Radiopharmaceutical Diagnostic Drugs: Nonclinical Study Recommendations, Guidance for Industry 2022), as outlined in Fig. 1. These studies, often termed as non-clinical or preclinical studies, include in vitro testing to elucidate and/or confirm possible mechanisms of action of the RP and performance of in vivo experiments in animals to establish the pharmacological and toxicological profile. Due to the ionizing radiation associated with RPs, their non-clinical evaluation also involves assessment of the radiation amount delivered to the various organs. The radiation deposited into organs (the absorbed dose, expressed in Gy) due to RP administration is termed dosimetry. While it is recognized that the results obtained during non-clinical studies cannot be fully translated to humans due to the inherent physiologic differences, nonetheless, these results provide an adequate estimation of the expected pharmacokinetic and toxicological profile. This is then applied as a supportive justification for demonstrating the minimization of the risk associated with RP use and also provides the basis for the selection of the scientifically justifiable administered activity [4). In the context of RPs, the term “dose” might also be employed describing the amount of administered radioactivity (expressed in Bq); moreover, depending on the intrinsic characteristics of the intended agent, its associated mass (in g or moles) may also be of concern (US Fda Guidance 2022).

RPs are distinctly different from conventional pharmaceuticals, nevertheless RP specific guidance documents are currently scarce (Schwarz and Decristoforo 2019) and may not cover all aspects necessary for the clinical translation of novel products. Hence, investigators and regulators often refer to existing guidance documents for non-radioactive drugs with limited usefulness and applicability for RPs. As a result, investigators engaged in the development of RPs often encounter uncertainties regarding the appropriate design of non-clinical studies, which carry the risks of collecting either an insufficient amount, irrelevant, or redundant data. One of the major sources of uncertainty arises from the general lack of a clear and harmonized regulatory framework, making it difficult and troublesome for both regulators and investigators to “navigate” through multiple guidance documents to extract information necessary for an appropriate study design. Moreover, due to the rapid expansion of molecular imaging and radioligand therapy, an ever-increasing variety of novel agents are being considered for their potential use in human subjects. These include functionalized and radiolabelled macromolecules, nanoparticles, or various ligands containing novel therapeutic radionuclides. As a result, the few existing RP-specific guidance documents risk to become unprecise or obsolete as soon as new technologies or applications are developed. For the above reasons, investigators are encouraged to have initial discussions with the regulators regarding a specific clinical trial to determine the most appropriate specific non-clinical study design. While this “case-by-case basis” approach is effective in some regions of the world with relatively well-developed and standardized regulatory structures, this remains a challenging problem in other areas where regulatory communication and training mechanisms between regulators and investigators are still developing.

## Main text

This document aims to highlight major regulatory challenges associated with designing and conducting non-clinical studies with RPs for subsequent clinical translation. In addition, we describe several of the key considerations that may aid both investigators and regulators in various regions of the world to address the challenges mentioned above.

Tables 1, 2 and 3 summarize main regulatory texts in non-clinical testing, dedicated to both non-radioactive Investigational Medicinal Products (IMPs) and RPs.

**Table 1:** Guideline documents from regulatory bodies on pharmaceuticals in general

Guideline/Text	Origin/Organization	Topic	References
ICH M3(R2): “Non-clinical safety studies for the conduct of human clinical trials and marketing authorisation for pharmaceuticals”	ICH (EMA, FDA)	General requirements on non-clinical safety studies	Anonymous (2009)
ICH S9: “Nonclinical evaluation for anticancer pharmaceuticals”	ICH (EMA, FDA)	Anticancer Pharmaceuticals	Anonymous (2010)
ICH S7A: “Note for guidance on safety pharmacology studies for human pharmaceuticals”	ICH (EMA, FDA)	General requirements on non-clinical safety studies	Anonymous (2001)
ICH S6(R1): “Preclinical safety evaluation of biotechnology-derived pharmaceuticals”	ICH (EMA, FDA)	Biotech pharmaceuticals	Anonymous (2011)
CHMP/SWP/28367/07: “Guideline on strategies to identify and mitigate risks for first in human and early clinical trials with investigational medicinal products”	EMA	General requirements on non-clinical safety studies	Anonymous (2018)
Directive 2001/83/EU “Community code relating to medicinal products for human use”	EU	“Pharmaceutical Directive” Requirements for toxicological and pharmacological studies	Directive (2001)
Directive 2010/63/EU on the protection of animals used for scientific purposes	EU	Animal welfare and protection	Directive (2010)
Directive 2004/2010/EC “Good laboratory practice: tests on chemical substances”	EU	GLP requirements	Directive (2004)
FDA/ICH Guidance Document: “Guidance For Industry, Co-development of Two or More New Investigational Drugs for Use in Combination”	FDA/ICH	Investigational Drugs	Research C for DE and. Codevelopment of Two or More New Investigational Drugs for Use in Combination [Internet] (2020)
FDA/ICH Guidance Document: “Redbook 2000:IV.B.1 General Guidelines for Designing and Conducting Toxicity Studies”	FDA/ICH	Toxicity Studies	Nutrition and for FS and A. Redbook (2000)
FDA/ICH Guidance Document: “Guidance for Industry, Investigators, and Reviewers: Exploratory IND Studies”	FDA/ICH	Exploratory investigational new drug (IND) studies	Research C for DE and. Exploratory IND Studies [Internet]. U.S. Food and Drug Administration (2019)

**Table 2:** Guideline documents from regulatory bodies concerning radiopharmaceuticals

Guideline/text	Origin/organization	Topic	References
EMA/CHMP/SWP/686140/2018: Guidance on non-clinical requirements for radiopharmaceuticals	EMA	Non-clinical requirements for radiopharmaceuticals–draft document	Guideline on the non-clinical requirements for radiopharmaceuticals [Internet] (2018)
Directive 2013/59/Euratom: BSS for protection against the dangers arising from exposure to ionising radiation	Euratom	Radiation Protection	EUR-Lex-32013L0059-EN-EUR-Lex [Internet] (2022)
Guidance on medical exposure in medical and biomedical research	EURATOM	Risk categories for radiation exposure in diagnostics	European Commission, Directorate-General for Environment NS and Civil Protection (1999)
EMEA/CHMP/QWP/306970 Guideline on radiopharmaceuticals	EMA	Quality requirements for radiopharmaceuticals when aiming for marketing authorization	EMA (2018)
FDA guidance 2011: nonclinical evaluation of late radiation toxicity of therapeutic radiopharmaceuticals	FDA	Therapeutic Radiopharmaceuticals	Research C for DE and Nonclinical Evaluation of Late Radiation Toxicity of Therapeutic Radiopharmaceuticals [Internet]. U.S. Food and Drug Administration (2020)
FDA guidance document: microdose radiopharmaceutical diagnostic drugs: nonclinical study recommendations, guidance for industry	FDA	Diagnostic Radiopharmaceuticals	US FDA Guidance. Microdose Radiopharmaceutical Diagnostic Drugs: Nonclinical Study Recommendations, Guidance for Industry (2022)
FDA/ICH Guidance Document: Oncology Therapeutic Radiopharmaceuticals: Non-Clinical Studies and Labeling Recommendations, Guidance for Industry	ICH (FDA/EMA)	Therapeutic Radiopharmaceuticals	US FDA Guidance (2022)

**Table 3:** Guideline documents on radiopharmaceuticals from professional organizations

Guideline/text	Origin/organization	Topic	References
Position paper on requirements for toxicological studies in the specific case of radiopharmaceuticals	EANM	Toxicity studies	Koziorowski et al. (2017)
EANM guideline for the preparation of an Investigational Medicinal Product Dossier (IMPD)	EANM	Guideline for chemical and pharmaceutical part of an IMPD for radiopharmaceuticals	Todde et al. (2014)
Guidelines to PET measurements of the target occupancy in the brain for drug development	EANM	PET occupancy study	Takano et al. (2016)
Guidance for preclinical studies with radiopharmaceuticals	IAEA	Technical consideration for in vitro and in vivo preclinical evaluation of radiopharmaceuticals	Guidance for preclinical studies with radiopharmaceuticals. IAEA radioisotopes and radiopharmaceuticals series (2021)
Acceptance testing for nuclear medicine instrumentation	EANM	Primarily for instrumentation in the clinics, but some instrumentation is also used in non-clinical setting	Busemann Sokole et al. (2010)
IAEA-TECDOC-1782 Good practice for introducing radiopharmaceuticals for clinical use	IAEA	Partly related to IMP/IMD dossier and nonclinical testing	International Atomic Energy Agency (2016)
International Atomic Energy Agency and World Health Organization guideline on good manufacturing practices for radiopharmaceutical products	IAEA	International GMP guidelines or radiopharmaceuticals	Annex 2 International Atomic Energy Agency and World Health Organization guideline on good manufacturing practices for radiopharmaceutical product [Internet]. WHO Technical Report Series (1025)(2020)

## Non-clinical pharmacology (pharmacokinetics, pharmacodynamics)

The general consideration as presented in ICH(M3) (Anonymous 2009) does not always apply to RPs, particularly diagnostic RPs, which are typically administered at microdose levels and are expected not to exert any pharmacological effect. Tests related to toxicity concerns are covered below in “Preclinical toxicology: from microdosing to a risk base approach” section. Non-clinical pharmacology studies should provide data to predict the human pharmacokinetics and pharmacodynamics of the RP. Although non-clinical in vivo studies in healthy animals and disease models are essential, results from various in vitro studies (e.g. in cells) should assist in the appropriate design of subsequent in vivo experiments, thus minimizing the number of animals used in these studies, under the 3Rs principle (Replace, Reduce, Refine; Directive 2010/63/EU) (Directive 2010).

### In-vitro testing considerations

#### Small molecules and peptides

It is essential to plan and design in vitro studies carefully before in vivo non-clinical testing. The in vitro study design should be based on the specific purpose of the RP in question, keeping in mind the final intended clinical application. Technical details on conducting in vitro tests are described elsewhere (Guidance for preclinical studies with radiopharmaceuticals. IAEA radioisotopes and radiopharmaceuticals series 2021; Decristoforo and Pfister 2021). Practical examples of the importance of in vitro testing can be found in most publications on diagnostic, therapeutic, and theranostic radiotracers when assessing e.g. the binding properties to a given target for better therapeutic efficacy and diagnostic accuracy (Kolenc Peitl et al. 2019).

In vitro non-clinical studies aimed to predict pharmacokinetics typically include in vitro serum stability, protein binding, and lipophilicity testing. In vitro serum stability testing is a relatively simple test providing initial information on metabolic stability. Unstable RPs will typically not proceed to clinical trials, unless a certain metabolic pathway is part of the mechanism of action (e.g. FDG). Additionally, there are emergent strategies to improve the in vivo stability of e.g. peptide-based RPs, therefore exceptions are possible in selected cases (Nock et al. 2014). The binding of the new RP to plasma proteins is typically included in non-clinical in vitro testing. Protein binding influences pharmacokinetics and may be predictive to demonstrate a potential level of accumulated background activity, which may impair the quality of the images for diagnostic applications. On the other hand, the introduction of albumin binders has been proposed for therapeutic applications of RPs, to increase protein binding in order to enhance the therapeutic effect (Lau et al. 2019). Another test usually included is the assessment of lipophilicity (logD_7.4_). Depending on the intended use of the novel RP, high or low lipophilicity may be desired. High lipophilicity is required for molecules intended to cross the blood brain barrier (BBB), while in most other cases higher hydrophilicity is preferable, to promote urinary excretion.

Non-clinical pharmacodynamic studies should address the mode of action and provide additional knowledge. The knowledge includes the interaction of the investigational RP with both target and non-target tissues, or cells. Different in vitro models provide the basis to evaluate the biological effects or molecular mechanisms of new drug candidates against specific cell lines and how these mechanisms may influence these cells under defined conditions. Usually, such studies include determination of binding profile and occupancy to receptors or enzymes, and functional consequences (agonistic/antagonistic; stimulatory/inhibitory), including appropriate cell signalling. Data obtained in this step helps to characterise the pharmacological effects (if any) and to identify the most relevant animal models. RP data on basic pharmacology can be obtained from publications on the same class of RP or on the unlabelled drug. A thorough literature search is important to reduce time and resources expended, the use of already established in vitro and in vivo models and following the 3Rs principle is highly recommended.

For most peptide-based RPs in vitro binding affinity and internalization rate studies are indicators of the pharmacology of the molecule under development, where the first parameter is needed to demonstrate the strength of the interaction between the target (i.e. receptor) and the vector, which in principle also indicates the final efficacy. It is recommended to provide comparative affinity data with a known compound interacting with the target in question, for example the unmodified peptide or a variant where data on human behaviour are available. In the development of peptide RPs often slight modifications, such as minor changes in the amino acid sequence or the chelator linked to the peptide, are performed. In such cases comparative affinity studies with the original compound are also required, to predict changes in targeting behaviour in vivo. Internalization rate or cell-associated rate is used as an indicator of cell bound radioactivity over time and is also an important parameter for predicting efficacy and the particular mode of action (e.g. agonistic vs antagonistic). Off-target effects can be indicated by in vitro binding studies on cells, which do not express the target in question or express different, possibly related, targets (e.g. another G-protein coupled receptor subtype).

#### Antibodies and other macromolecules

As opposed to low molecular weight molecules and peptide-RPs, macromolecules, like radiolabelled antibodies, represent a rapidly expanding class of compounds, usually in the form of a radionuclide coupled to a biologic system-derived ligand such as a full-size antibody or an antibody fragment. Typically, at first an unmodified antibody-based compound is selected, for which a complete data set of non-clinical studies has been generated and which is available in the quality appropriate for biologic therapeutics. Therefore, clinical development relies heavily on the availability of fully characterized Good Manufacturing Practice (GMP)-grade “precursor moieties”. The discussion is then more focused on the possible changes in pharmacology due to protein functionalization (e.g., bifunctional chelator attachment) aimed to subsequent radiolabelling, taking into account the clinical study design factors such as the number of administrations, total mass dose, possibility of immunogenic response, etc. It should be recognized that the unmodified antibody itself provides the most significant potential impact on the radiolabelled antibody pharmacology and toxicity potential in vivo. Therefore, in the presence of adequate non-clinical data or prior clinical experience with the unmodified antibody, the supplemental non-clinical evaluations of the radiolabelled antibodies could be limited to in vitro and in vivo determinations to ensure that the critical characteristics such as antibody potency, monomer content, and biodistribution are preserved. For example, bioconjugated antibodies, mini- or nano-bodies, and other macromolecules need additional in vitro testing to determine whether random labelling with zirconium-89, copper-64, yttrium-86, gallium-68 or other radionuclides significantly affects the binding properties at the binding surface of the antibody (Sharma et al. 2019). Additional testing with ELISA, surface plasmon resonance, circular dichroism (conformational changes), and selectivity towards other targets are usually included in the in vitro non-clinical assessments (Pretto and FitzGerald 2021), besides stability and radiotoxicity. Furthermore, prior preclinical and clinical experience with the unmodified antibody, if available, may be used as justification for establishing the total dose range to be evaluated during the optimal dose-finding phase I study. In certain circumstances (e.g., when extensive clinical data exist for the unmodified antibody and the same antibody that has been modified with other chelators), the regulators should be able to rely on a scientifically justified risk-based analysis to determine whether an additional new chelator-specific non-clinical evaluation is warranted.

Lastly, it should be recognized that no relevant regulatory guidance may exist for completely novel RPs with very limited or emerging availability (e.g., radiolabelled liposomes or nanoparticles). In those situations, the exact study design should be based on the available scientific knowledge of the investigational agent properties. The investigators are encouraged to collect and present as much critical data as possible pertaining to the RP itself and its expected behaviour in humans, to agree to the particular non-clinical evaluation studies design.

More specific information on required in vitro testing can be found in the recently published IAEA technical document Guidance for preclinical studies with RPs (Guidance for preclinical studies with radiopharmaceuticals. IAEA radioisotopes and radiopharmaceuticals series 2021).

### In vivo* testing considerations*

In vivo studies with animal models provide the closest non-clinical link towards the clinical translation of new drugs and radiotracers. The Food and Drug Administration (FDA) and the European Medicines Agency (EMA) require extensive small animal preclinical testing (e.g., rodents). Only in rare cases testing with larger animals is recommended (Anonymous 2009), for instance in selective cases in neurological applications as further elaborated in “Neurological applications” section. For RPs, certain studies may not be required, such as in cases where the administered mass is low or negligible (see “Preclinical toxicology: from microdosing to a risk base approach” section).

Again, it is worth mentioning that when testing new RPs in vivo, it is critical to design the study carefully, keeping in mind the intended clinical use (Henderson et al. 2013) and compliance with the 3Rs principle. (Directive 2010) This means that precise biostatistical power analysis may be required and retrospective study design to reduce the need for animal testing. Regarding clinical translation, the following studies on healthy animals may be considered or recommended by regulatory agencies: biodistribution, dose-escalation, dosimetry, pharmacokinetics (clearance, elimination pathways), pharmacodynamics (compartmentalization, uptake, reactivity, transformation) or ex vivo analysis (staining, omics, metabolism). (Guidance for preclinical studies with radiopharmaceuticals. IAEA radioisotopes and radiopharmaceuticals series 2021) Additional toxicity testing in animals is required, as outlined in “Preclinical toxicology: from microdosing to a risk base approach” section. In vivo imaging studies play a significant role at this stage in predicting the therapeutic/diagnostic outcome of the RPs (see “Imaging” section).

We also recommend careful selection of the animal models for the non-clinical in vivo testing of RPs (animal background, immune-deficient, immune-compromised, genetically engineered, species differences) so that testing criteria can be matched and standardized.

In vivo pharmacokinetics is one of the fundamental studies included in the non-clinical testing of RPs. Biodistribution studies in a suitable animal model are needed to demonstrate target vs. non-target radioactivity uptake and retention (which may or may not be complemented by imaging studies). Authorities often require data on “off-target” effects, which may be addressed by including blocking studies in the experiments to show specific interactions e.g. in healthy tissue. Healthy mice are typically used to assess the physiological distribution of the RP, which can also be exploited for dosimetric calculations (see also “Non-clinical dosimetry” section). Quantitative imaging is increasingly used in biodistribution studies (see “Imaging” section). Specific considerations for RPs in neurology are addressed in “Neurological applications” section. More technical information on the conduction of in vivo tests is provided in Guidance for preclinical studies with radiopharmaceuticals. IAEA radioisotopes and radiopharmaceuticals series (2021).

## Non-clinical safety-radiation effects

### Non-clinical dosimetry

Small animal dosimetry data are essential in the development phase of new RPs. Depending on the objective, two broad applications can be considered:

- Development of new diagnostic tracers
- Dosimetry for molecular radiotherapy

In the development of novel diagnostic tracers, regulatory agencies (e.g. FDA, EMA) require information on the order of magnitude of the absorbed dose delivered to humans. This is done by “reference dosimetry” comparing the irradiation delivered by various RPs (McLaughlin 1989).

The European Commission has issued a guidance document to define risk categories for medical and biomedical research based on effective doses delivered (Table 4) (Commission and Directorate-General for Environment NS and Civil Protection 1999). In that context, small animal experiments are performed to derive pharmacokinetics (PK) parameters (usually mice). This information is used to extrapolate PK to humans (allometry) for integration into human dosimetric models. There are ongoing discussions that such extrapolation is of limited value when it comes to the application of very short lived diagnostic radionuclides (e.g. C-11) (Zanotti-Fregonara et al. 2021).

**Table 4:** Risk categories dependent on effective dose. Limits valid for population < 50 years, excluding paediatrics

Benefit	Risk	Risk category	Probability	Effective dose [mSv]
Low	Not significant	I	≈ 10^–6^ or less	< 0.1
Moderate/medium	Intermediate	II		
		IIa	≈ 10^–5^	0.1–1
		IIb	≈ 10^–4^	1–10
Significant	Moderate	Cat. III	≈ 10^–3^ or more	> 10

For paediatrics, limits have to be decreased by a factor of 2–3. For population > 50 years, limits may be increased by a factor of 510 (from Commission and Directorate-General for Environment NS and Civil Protection 1999)

The determination of time-activity curves (TAC) is usually obtained from animal groups, based on the conventional “cut and count” approach. Longitudinal studies based on quantitative imaging of small animals are increasingly applied to decrease the number of animals, whereby accuracy depends on the applied standardization. (see “Image-derived dosimetry studies” and “Activity determination and dosimetry standardization” sections).

The extrapolation of pharmacokinetic data (TAC, time-integrated activity or time-integrated activity coefficient) from the animal to the human is not trivial. RP design often assumes that relative radioactivity concentrations in organs (%IA/g) between animal and human are proportional to their whole body mass ratio (McParland 2010).

The way extrapolation is performed and the hypothesis selected are rarely documented in published literature. Additionally, a systematic review of the suitability of the extrapolation (post hoc, during clinical studies) is usually not performed. There is a pressing need for standardisation in the aforementioned areas, thus guidance on procedural reporting should be generated.

Absorbed doses are computed following International Commission on Radiological Protection (ICRP) recommendations. Most existing reports on RP dosimetry present data computed according to ICRP 60 (Smith 1991) recommendations. Even though the recent ICRP 103 (Good Laboratory Practice (GLP)-OECD [Internet] 2021) was published 15 years ago, applying it to RP dosimetry is quite demanding. ICRP 103 uses voxel-based computing models for absorbed dose calculation instead of the mathematical one used in ICRP 60. Changes have been made in tissue weighting factors used in effective dose calculations and the calculation framework has changed as it requires the computation of male and female organ absorbed doses separately. Some codes have been proposed for this task (Andersson et al. 2017; Stabin and Farmer 2012), but most PK studies consider only pooled values (obtained for both male and female patients).

In conclusion, the implementation of ICRP 103 recommendations in clinical practice (for example during the development phase of a new RP) is still a complex procedure requiring some practical implementation guidelines. Therefore, today the main needs related to small animal dosimetry are the development of solid recommendations and procedures that allow the standardisation of practice.

### Specific tests for therapeutics

Targeted Radionuclide Therapy (TRT) is gaining momentum, with the recent approval of [^177^Lu]Lu-DOTATATE (LUTATHERA^*®*^) and the development of [^177^Lu]Lu-PSMA-617 (PLUVICTO^®^) (Sgouros et al. 2020). Alpha- (e.g. ^225^Ac or ^211^At) and Auger electron (AE) emitters are emerging as attractive therapeutic alternatives (Ku et al. 2019). The non-clinical assessment of a potential therapeutic RP should include additional in vitro and in vivo assays to predict the efficacy and potential toxicity of the therapeutic application. This may include detailed cellular assays (using 2D and/or 3D models) and studies with adequate cancer animal models, as well as “mouse-specific” dosimetry. The type of studies will depend on the type of emitted radiation and is also related to the particular application of the radiotracer under evaluation. In many cases it will not be possible to do all investigations before first in human applications. The FDA provides specific guidance to the conduct of non-clinical studies of therapeutic RPs for oncology (FDA 2020) and recognizes that certain studies e.g. the investigation of late radiation effects may be performed at later stages of clinical development, i.e. Phase II or III studies (Research C for DE and Nonclinical Evaluation of Late Radiation Toxicity of Therapeutic Radiopharmaceuticals [Internet]. U.S. Food and Drug Administration 2020).

#### Cellular studies

##### Cellular uptake and subcellular localization

Besides binding affinity measurements, cellular uptake, internalization, and blockade studies, the preclinical evaluation of therapeutic radioconjugates may also involve the determination of the spatial distribution of the radionuclides inside the tumour cells. Especially in the case of alpha- and AE-emitters, to better account for the observed radiobiological effects due to short range particles. The subcellular distribution can be determined mainly using fractionation assays and micro-autoradiography (Guidance for preclinical studies with radiopharmaceuticals. IAEA radioisotopes and radiopharmaceuticals series 2021; Bavelaar et al. 2018).

##### Radiobiological effects

Cellular assays must focus on the evaluation of the cytotoxicity of therapeutic RPs under study, upon exposure of the target cells to increasing activities of the compound. This assessment is usually performed based on viability and clonogenic assays (Buch et al. 2012).

Nuclear DNA is the primary target for ionizing radiation, as the irradiation of the cells can induce DNA damage indirectly via water radiolysis or directly by one-electron oxidation. These processes can result in DNA single-strand and double-strand breaks (DSBs), as well as DNA crosslinks and DNA base damage. Unrepaired damage leads to cell death by mitotic catastrophe or apoptosis. Thus, it is vital to assess DNA damage and apoptotic outcome in tumour cells treated with therapeutic RPs. DNA damage can be evaluated using various techniques, as described elsewhere (Bavelaar et al. 2018; Mah et al. 2010; Subiel et al. 2016).

#### Animal studies

##### Biodistribution, pharmacokinetics and radiotherapeutic assays

The biodistribution and pharmacokinetics of therapeutic RPs should be studied in adequate cancer animal models (e.g. subcutaneous or orthotopic tumour-bearing mice) as described elsewhere with, in principle, no difference to diagnostic RPs. For this purpose, after confirmation of tumour induction, in vivo imaging studies might be combined with ex vivo dissection and counting studies as discussed in “Specific tests for therapeutics” section.

The long-term radiotherapeutic assays (several weeks/months depending on the treatment outcome) involve the administration of single or multiple doses of therapeutic RPs under study. Tumour volume measurement and monitoring of animal body weight should be carried out throughout the experiments to estimate the therapeutic efficacy and potential toxicity issues.

To better understand the radiotherapeutic effects and to support non-clinical data, additional studies may be helpful, e.g., excision of tissues from the tumours at given time points. This evaluates the intratumoural distribution of the radionuclides and confirms radiobiological effects derived from the in vitro cellular studies.

##### Dosimetry studies

“Mouse-specific” dosimetry is relevant, in the context of non-clinical molecular radiotherapy, to evaluate radiation effects induced in the animal during therapeutic procedures. The concepts developed for clinical dosimetry apply in the sense that model-based and specimen-specific dosimetry can be considered, albeit with specificities related to the scale of the dosimetric problem. It must be stressed that even though the administered activities can somehow be scaled by the mass of the animal (by a factor of 3000 between a 25 g mouse and a 75 kg human), the radiation range of the isotopes considered remains the same, and this has consequences on the results obtained. Standardisation is also largely missing in this domain, limiting the possibility to compare results obtained in different experiments (Mauxion et al. 2013).

## Preclinical toxicology: from microdosing to a risk base approach

### ICH guidelines M3(R2)

Preclinical toxicological assessment is part of the general experimental testing aimed to provide non-clinical information and general guidance on pharmaceuticals is provided by “ICH guidelines M3(R2) on non-clinical safety studies for the conduct of human clinical trials and marketing authorization for pharmaceuticals” (Anonymous 2009), which has long been the only reference document for RPs related clinical trials as well. The goal of the above guideline is to evaluate potential toxic effects to target (and non-target) organs, estimate initial safe dose and dose range, and identify parameters to monitor potential adverse effects. The document embraces most of the possible situations and levels of attention, and it also includes indications about the determination of genotoxicity, carcinogenicity, and more. Different approaches are listed in a dedicated Table (No 3 in the document). The microdosing concept has been introduced, aimed to help in exploratory clinical trials (early-phase clinical trials) and to speed up and promote trials themselves, reducing time and related costs. Here, two different approaches are described for microdosing: i) microdose is a total dose < 100 µg, which is at the same time ≤ 1/100 of “no observed adverse effect level” (NOAEL) and ≤ 1/100 of pharmacologically active dose; ii) a cumulative dose < 500 µg, with a maximum of 5 administrations with washout between doses and every single dose still complying with the above requirements (< 100 µg, ≤ 1/100 of NOAEL, etc.).

### EMA guideline on the non-clinical requirements for RPs

In 2018 EMA drafted the “Guideline on the non-clinical requirements for radiopharmaceuticals” (Guideline on the non-clinical requirements for radiopharmaceuticals [Internet]. 2018) which has not been finally adopted yet. It deals with the non-radioactive part of an RP and the evaluation of its pharmacological and toxicological effects and recognizes that major risks of RPs are related to radioactivity, which require different methods (mainly dosimetry). One of the goals of the guidelines is to reduce the extent of animal experiments (Directive 2010), while it also attempts to propose a better, moreadaptable regulatory framework by considering specific peculiar characteristics of RPs. To this regard, four different scenarios are depicted, depending on the extent of “novelty” of the non-radioactive part of the RP, which has correctly been indicated as the target of non-clinical testing. Pharmacology and pharmacokinetics are common to different scenarios, the major difference being in toxicological requirements.

#### *Scenario* 1

The candidate RP is almost identical to an already known radiolabelled molecule, the only difference being the radionuclide. An example may be a RP derived from the well-established [^68^Ga]Ga-DOTATOC, where another radionuclide replaces gallium-68 (e.g. scandium-44 or copper-64); in this case it is supposed that in vivo behaviour of the new RP is not significantly different from that of the “parent” compound, and valuable data for a clinical trial application may be inferred by previous applications related to the same non-radioactive part. Good quality scientific literature can also be used. If no reference data are available, biodistribution studies with single application need to be performed, with minimal histopathology examinations.

#### Scenario 2

The radionuclide is added to a known pharmaceutical (e.g. [^18^F]fluoroestradiol to estradiol) or a small functional group or a linker is added to a large molecule, such as a protein. Here it is important to demonstrate that introducing the radionuclide/functional group does not significantly alter the pharmacokinetic properties of the “cold” part of the RP; one of the two above described microdose approaches are requested, the choice of which depends on the expected kinetic (fast or slow) of the RP. In this case, molarity needs to be taken into account; for example, a sample of a peptide with a molecular weight of about 3000 and potency comparable with that of a smaller molecule (e.g. MW = 300), would have 1/10 of molarity, which should be the correct reference parameter to be considered; thus, a mass > 100 µg could be accepted, although the guideline does not provide a precise limit. Experiments should be conducted in one species only, and haematology, histopathology and clinical chemistry should be evaluated. No genotoxicity studies are requested, and no significant differences are foreseen for diagnostic or therapeutic RPs, except for dosimetry studies, which should be performed in an animal model of disease for therapeutics.

#### Scenario 3

If the expected dose is > 100 µg, toxicity studies are in principle as described above for the 2nd scenario, except for the need to conduct studies in two different animal species if the RP shows pharmacological activity at the intended dose and investigation of tolerance should also be performed, if applicable, again. In the case of therapeutics, dosimetry studies should be performed in an animal model of disease; genotoxicity has to be evaluated (usually Ames testing).

#### Scenario 4

The last scenario is related to a RP to be administered in multiple dosing. Theoretically, it is applicable to both diagnostic and therapeutic RPs. However, such a situation is more frequent for therapeutics. Toxicity studies should be performed with two different animal species, except when no pharmacological activity is displayed at the intended dosage; investigation of tolerance and standard core battery for safety pharmacology should be performed. The Ames test is often required, but can be omitted in the therapy of advanced cancers, where ICH S9 guidelines (Anonymous 2010) are applicable. Carcinogenicity and reproductive tests may also be omitted for therapeutic RPs.

Finally, the EMA guideline states that compliance with Good Laboratory Practice (GLP) is not requested for the RP itself. However, it remains necessary for the non-clinical toxicology of the “non-radioactive” part, which is a significant difference with the US approach, where conduction of the studies in a comparative anatomy or veterinary university medicine department is allowed (see also “Good laboratory practices (GLP)”section).

### Other recommendations

In the meantime, the FDA released a guidance document dedicated to diagnostic RPs, which was intended to help Marketing Authorization (MA) applicants in developing suitable strategies for microdosing (Microdose Radiopharmaceutical Diagnostic Drugs: Nonclinical Study Recommendations [Internet] 2018). Interestingly, the US Agency fully recognizes the broad range of different possible RPs, and clearly states that toxicity testing may not always be necessary, granting a waiver if justified. The above guidance follows the same general principles defined in ICH M3(R2) document, but with some useful differences: (i) genotoxicity studies are usually not requested, based on the single exposure with microgram quantities principle, and the statement applies to any phase of the proposed clinical trial; (ii) the same applies for safety pharmacology, investigation of maximum tolerated dose and repeated dose toxicity studies; (iii) a limit for protein products of ≤ 30 nmole is set.

A different approach has been proposed by the EANM Radiopharmacy Committee in a position paper released in 2016 (Koziorowski et al. 2017), which underlined that subacute and chronic toxicological studies, teratogenicity, genotoxic, and carcinogenic studies are usually not of concern for RPs, as exposure is often limited to a single dose (at least for diagnostic RPs). Moreover, RPs are usually not given to pregnant women. The paper considered different scenarios, proposing a toxicological limit of < 1.5 µg in case the desired RP is obtained via an efficient purification step, which allows removal of any chemical, radiochemical and radionuclidic impurities, including any precursor, quantitatively. In this case the “cold” counterpart of the RP could be used for a toxicological testing purpose (e.g. [^19^F]F-PSMA for [^18^F]F-PSMA). This might also be avoided, based on case-by-case risk analysis and relevant data from previous applications or scientific papers. The proposed limit is, in turn, based on the “threshold of toxicological concern” (TTC), which is a value associated with acceptable risk even in the worst case of genotoxic impurities. The above limit would not apply in case of known and highly toxic compounds and/or functional groups, such as nitrosamines. The second limit proposed by the authors is the same as stated by ICH M3(R2) guidelines for microdosing approach (< 100 µg), and an extended single dose study should be sufficient, especially if data obtained by biodistribution studies are already available (which is often the case). Finally, in case no purification steps are feasible and all the reaction mixture is administered to the patient, such as for instance when peptides are radiolabeled by complexation with a radiometal (e.g. [^68^Ga]Ga-DOTATOC), > 100 µg are injected, and approach 3 of ICH M3(R2) would apply, with toxicological studies performed on two different (rodent and non-rodent) species, and also the Ames test for genotoxicity is needed; for therapeutic RPs, the ICH S9 guidelines (Anonymous 2010) also apply, and for RPs aimed to treat oncological diseases genotoxicity test for phase I and II studies can be omitted.

### The risk-based approach

**Fig. 2:**
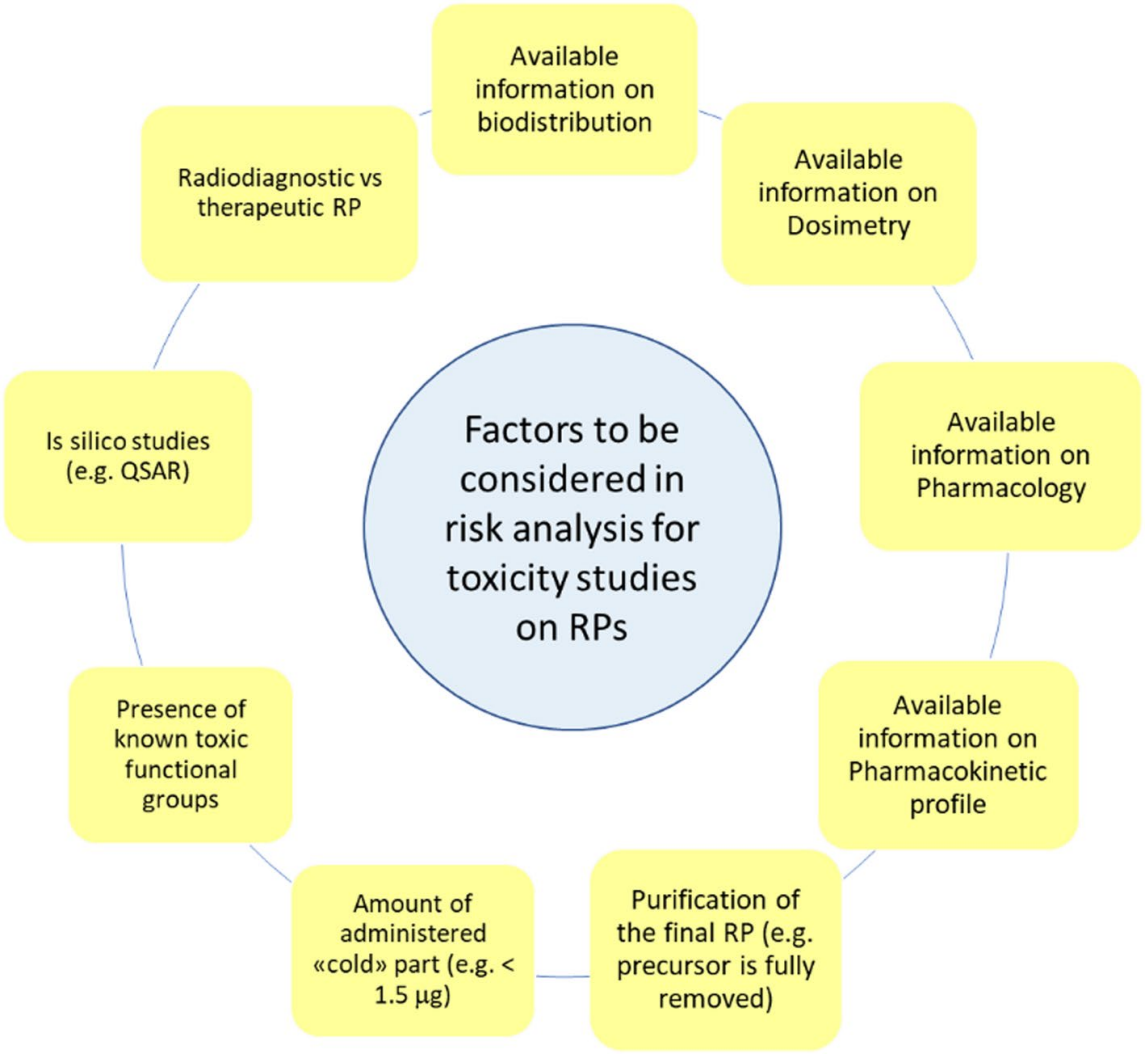
Factors to be considered in the risk analysis for toxicity studies of RPs

From the above discussion it is clear that several types of RPs exist, with a broad range of mass amounts of “cold” compound administered, based on a wide variety of preparation procedures (and for many different clinical applications), and there is no unique approach to toxicological studies. Thus, a risk analysis has to be performed every time a clinical trial involving the use of RPs is proposed, based on the following factors (see also Fig. 2): (i) the availability of biodistribution, dosimetry, and any other data related to the “cold” part of the RP; data may be obtained from MA application or other pharmaceutical dossier, if permission is granted, or by the scientific literature in case of well-established RPs; (ii) pharmacology information, via in vitro binding affinity studies, (iii) the expected pharmacokinetic profile, which can be obtained after suitable in vitro and in vivo studies on animals; (iv) the preparation procedure, i.e. whether final product is obtained after a suitable, efficient purification or the entire content of the reaction mixture is administered to the patient; (v) the presence, if any, of functional groups known to increase toxicity; (vi) in silico studies which, although not sufficient, may provide helpful prediction about toxicity; (vii) whether the desired RP is diagnostic or therapeutic.

In conclusion, risk assessment may be a very useful tool to drive the choice of type and level of toxicological studies to be performed. Considering the high related costs of these tests, it might significantly help to expand the number of applicants and increase the pace of new useful RPs to be assessed in clinical trials, thus translating the many exciting findings coming from research to the final patients. It is of paramount importance to involve and discuss with regulatory authorities, in an attempt to promote and translate the above-depicted more flexible approach into reference guidelines.

## Specific considerations

### Imaging

Although RP development has primarily relied extensively on ex vivo techniques, which use metric scales, and in some cases are more coherent between animals and humans (Blanchard and Smoliga 2015), many regulatory agencies now expect the inclusion of in vivo non-clinical imaging data and cross-validating multimodal methods for accessing biological processes. Over the last decade, novel imaging solutions and agents, capable of providing valuable information on the biological processes under study, have become readily available. Also, dedicated scientific and imaging instrumentation keeps improving, reaching higher and higher levels of accuracy, sensitivity, and resolution (Amirrashedi et al. 2020). It has been demonstrated that in vivo imaging techniques can very efficiently cover most studies, resulting in better in vivo assessment of pharmacologic and therapeutic effects of therapeutic agents especially at the very early stages (Lauber et al. 2017; Koo et al. 2006). The key aspects of biodistribution, pharmacokinetics and spatiotemporal receptor binding profiles of candidate biomolecules can be addressed efficiently and effectively through imaging methodologies and workflows (Koo et al. 2006; Rouchota et al. 2021) with only a fraction of the number of animals that would be needed for ex vivo biodistribution studies.

#### Image-derived biodistribution studies

Biodistribution in relevant species is among the most basic information in preclinical imaging. The studies can identify the potential target organs and tissues and demonstrate which on-target or off-target effects might be expected. The RP takes part in physiologic processes according to its pharmacological properties. The concentration in the body tissues is thereby dynamically changing. In dynamic imaging, the image sequence is started at the time of injection, and uptake, metabolism, and excretion of the RP are followed by collecting counts in intervals from seconds to minutes. Tissue-specific volumes of interests (VOIs) are manually drawn on the image, and associated activity is calculated. Organs selected include heart, brain, kidney, liver, spleen, bladder, muscle, bone, etc. Temporal biodistribution profiles may be used to address kinetics and reversibility of target- and/or off-target-mediated accumulation. Comparison of temporal biodistribution profiles between the genetically engineered and wild-type mouse strains or between the disease models and healthy animals may provide helpful insight on sites and kinetics of target-mediated elimination. Differences in biodistribution of a group of RP candidates for the same target can be used to determine a competitive advantage for one drug versus another (Loudos et al. 2019).

#### Image-derived dosimetry studies

Non-clinical dosimetry requirements are described in “Non-clinical dosimetry” section. Accurately estimating the tracer kinetics for dosimetry purposes typically requires a different protocol to that generally used in dynamic PET and SPECT imaging. To sufficiently describe the kinetics, image acquisition starting times of 1/3, 2/3, 3/2, 3, and 5 multiples of the effective half-life are recommended according to the International Commission on Radiation Units, and Measurements report no 67 (Report 2022). In the case of radionuclides with longer half-lives, even F-18, this necessitates multiple scanning sessions. The images of numerous studies may be separated by hours or even days depending on the half-life of the isotope in question. The first task is to combine those images into a consistent dynamic series. A crucial element during data merging is the proper handling of decay correction for independently acquired series. Automated software fusion techniques allow for more accurate anatomic correlation of functional/metabolic findings and have significantly improved the specificity and accuracy of PET and SPECT.

#### Multimodality imaging

Multimodal imaging increases the robustness of non-clinical imaging applications, as the possibility to gather information from complementary modalities. Magnetic resonance (MR) imaging, PET, SPECT, optical imaging (OI), and computerized X-ray tomography (CT) are among the most useful modalities. A practical example of this complementarity has been reported in many studies about tissue connectivity and associated functional effects from developed RPs.

A variety of dedicated small animal imaging systems are commercially available today (Kiessling and Pichler 2011). This includes standalone PET SPECT and CT systems, hybrid PET/CT, PET/SPECT/CT, SPECT/CT, SPECT/MR, PET/MR systems and simultaneous PET/MR systems. The primary rationale for combining PET and SPECT with CT or MR is to add anatomical information. CT is especially good for imaging bone and lung tissue whereas MR provides excellent soft-tissue contrast and multi-parametric MR can add significant morphological and functional information.

In pnon-clinical oncology applications, MR offers the unique ability to detect tumour margins/volumes in a broad range of models, improving the functional analysis of complementary PET data. Owing to heterogeneous radiotracer uptake in some tumours, PET tracer signal and thresholding will not always provide a reliable volume for such calculations. As a result, MR is useful for assessing partial volume effects, visualizing accurate tumour margins, and evaluating the tracer distribution within individual tumours to generate desired VOIs and calculate Standardized Uptake Values (SUVs) based on experimental objectives, obviating the need for post mortem studies. Further, high confidence data can be obtained through a longitudinal time course. In addition to the multi-parametric information MRI can provide on top of morphological information, simultaneous PET/MR has gained popularity in clinical imaging studies because it can reduce the time the patient is in the scanner. For non-clinical applications it is maybe less important but its high time efficiency for acquiring PET and MRI information at the same time can be important for high throughput imaging and for studies when the temporal correlation of PET and MRI information is essential, i.e. when observing instant drug/tracer effects or when using MRI based input function in PK/PD PET studies.

For precise PET quantification attenuation correction (AC) is needed. Attenuation of the PET signal can come from the animal tissue (larger animals and areas of bone structures) animal cradles, monitoring accessories and MR-coils (only relevant for simultaneous PET/MR). AC using CT is the gold standard whereas it is currently being validated for PET/MR. This should be taken into consideration when planning non-clinical imaging studies. In addition, non-clinical protocols should be optimised by defining the best compound concentration, animal preparation, administration and other critical parameters such as keeping the animals physiological parameters stable during scanning, since this can have a considerable effect on the robustness, accuracy, required time, resources, and ethical aspects of non-clinical research.

Researchers should consider both performance of specific hybrid technologies and specific hardware and software workflow implementations to account for unique aspects of multimodal detections. It is recommended to choose a non-clinical imaging system with similar performance characteristics as the clinical imaging system that will be used. This can boost a shorter preclinical evaluation period and thus accelerate the translation of new drugs to clinical practice.

**Fig. 3:**
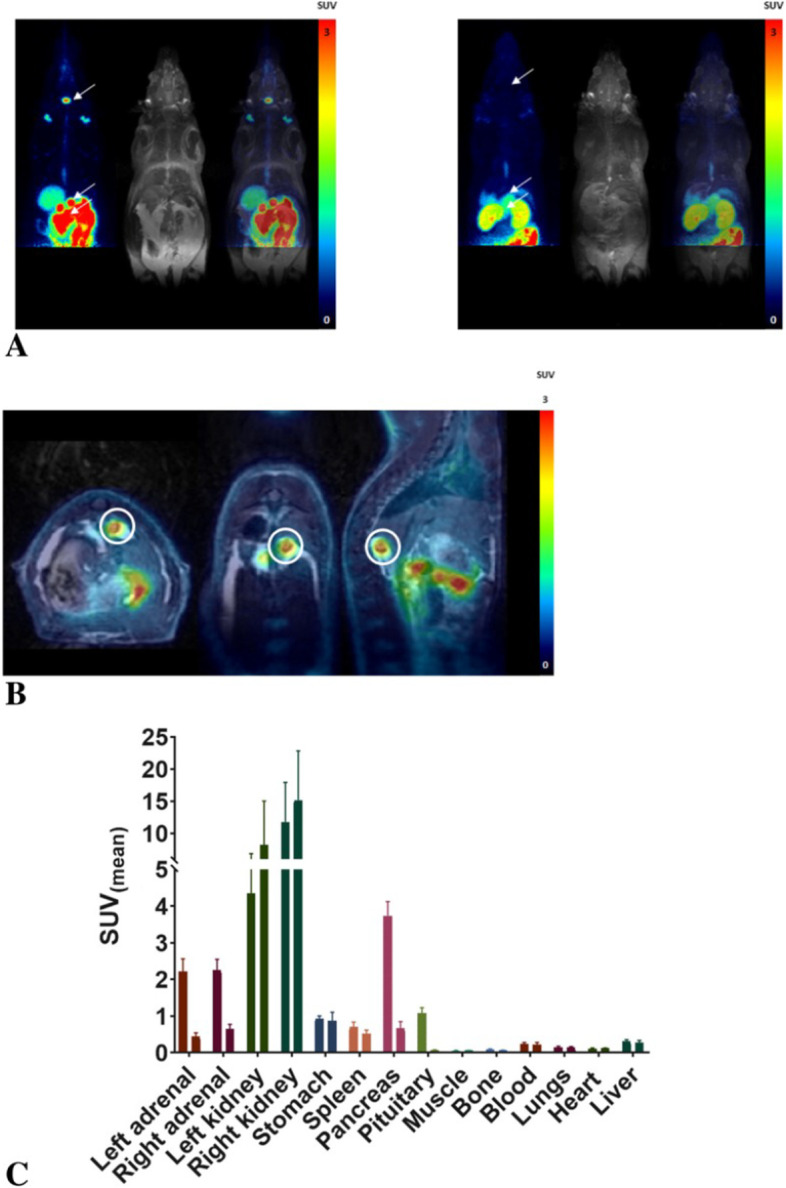
Example of multimodality PET/MRI imaging providing several advantages in the context on non-clinical testing of radiopharmaceuticals. The use of anatomical information from MRI provides multiparameter longitudinal assessment in a preclinical model (**A**: native image (left) and blocked with excess target ligand (right)) with superior mapping of organs **B** for longitudinal biodistribution/blocking studies and delineation of uptake despite influence from renal excretion. Additionally biodistribution is calculated from the imaging data **C** (from Tshibangu et al. 2020, legend modified)

Figure 3 gives an example of the use of multimodality imaging in radiopharmaceutical development.

#### Quantitative imaging and pharmacokinetic modelling

Most quantitative imaging approaches are based on PET, whereby quantitative SPECT is gaining momentum. Non-clinical quantitative PET provides valuable information of new PET ligands as it confirms target engagement, elucidates on binding characteristics and whether these should be considered reversible or irreversible, and helps estimating clinical PET acquisition intervals needed for accurate and precise quantification in humans. Therefore, the specific binding of the PET tracer to the target of interest or the flow through a biochemical pathway must be quantified to better estimate in vivo target expression levels or underlying physiological tissue parameters of relevance. To achieve accurate PET quantification, one should try to account for factors other than specific tracer binding contributing to the PET signal.

A straightforward approach to account for the differences in animal weight and administered tracer activity is using SUVs (Lodge 2017). However, several factors can significantly impact on the variability of SUV (Huang 2000), and therefore its use should be carefully considered (Hildebrandt et al. 2008; Vanhove et al. 2015). For oncological PET tracers, SUV ratio (SUVR) is often considered relative to muscle and liver to calculate tumour-to-normal tissue or tumour-to-background ratio. In addition, a tumour-to-blood ratio can be considered, predominantly if the blood activity concentration can be determined using the heart cavity or frontal lobe of the liver. For an SUV or SUVR approach, scanning is limited to a single late time point, although this time point needs to be chosen carefully based on the transient or pseudo-equilibrium (Carson et al. 1993; Ito et al. 1998).

In addition, dynamic imaging can provide valuable information on tracer binding characteristics with displacement or chase experiments where cold ligand or competing drug is administered during the dynamic PET scan. This way, reversible or irreversible kinetics can be confirmed. In addition, specific tracer binding can be demonstrated by faster tracer clearance from tissue or reduced PET signal after pre-treatment with a drug with high affinity and selectivity for the same target as the PET tracer. Although these experiments are more observational than quantitative, they can support the validation of new PET tracers without being technically challenging.

Dynamic imaging can also be combined with an appropriate input function to apply kinetic modelling (Gunn et al. 2001) to determine quantitative endpoints such as distribution volume or binding potential for reversible tracer kinetics (Innis et al. 2007; Logan 2000) or the net influx rate constant (Ki) for irreversible kinetics (Patlak et al. 1983). The input function can be determined either from a suitable reference tissue (Logan et al. 1996; Lammertsma and Hume 1996) or by arterial blood sampling (Mann et al. 2019). However, this approach is logistically challenging and not suitable for high throughput imaging.

Overall, the added value provided from the non-clinical imaging towards clinical translation is undeniable. However, state-of-art non-clinical and clinical imaging technology with high-throughput analysis requires a substantial investment (financial and logistic), which is obviously a barrier when disseminating and standardizing imaging methods. Thus, the scientific community (academia and industry) must direct efforts towards establishing a solid network for testing new RPs in regulated facilities to make advanced imaging widely accessible and promote standard assessment procedures.

### Neurological applications

The development of RPs that target brain disease and function usually possess a different physiochemical property profile compared to other RPs. This is mainly due to the blood–brain-barrier (BBB), which displays an insurmountable hurdle for many chemical entities to enter the brain. Therefore, specific non-clinical tests have to be included to provide data for BBB penetration. These tests are usually carried out in healthy control animals and in respective disease models. In most cases, this includes imaging and ex vivo studies using experiments as described in an IAEA guidance (Guidance for preclinical studies with radiopharmaceuticals. IAEA radioisotopes and radiopharmaceuticals series 2021).

Many nanomedicines (e.g. mAbs, NBs, polymers, etc.), peptides or small molecule-based drugs cannot pass the BBB, either due to their size or polarity, or both. At first sight, there are not many differences among documents that should be provided to the authorities to get approval for clinical studies. Information about efficacy and safety is key in this respect, as discussed in the previous sections. However, the rise of new techniques to guide RPs into the brain brings another layer of complexity into the application process, as these techniques could also display toxicology concerns which may eventually outweigh the advantages. Frequently used technologies are focused ultrasound (FUS) in connection with microbubbles (Meng et al. 2021), chemically-induced BBB opening with drugs such as mannitol (Lesniak et al. 2019), or receptor-facilitated transport hijacking, as in the case of the transferrin receptor (Sehlin et al. 2016). All these techniques bring additional challenges, as they can damage or even destroy cells through high energy deposition, additional surgery risks or additional chemicals required to be used. The first clinical application of FUS-mediated delivery of ^111^In-labeled trastuzumab was published just recently (Meng et al. 2021). However, future studies are still needed—not only for FUS, but also for all technologies mentioned above—to proceed as standard operations without concerns. Risk-benefit estimates need to be carried out and future non-clinical studies should instead focus on these aspects rather than exploring the technology to target just the next protein beyond the BBB.

As a general remark, and independently of the application to translate RPs from non-clinical to clinical studies, it is important to remember that species differences can have a massive influence on the possibility of an RP reaching targets within the brain. For example, rodents possess a higher efflux transporter activity than larger species (Syvänen et al. 2009). Therefore, low brain uptake could be species-specific and uptake in rodents might not be predictive of that in humans. Anaesthetics may also have a negative impact on the results. A second species (e.g. pig, dog or non-human primate) or other measures to test for species differences may be used to validate the results obtained in rodents (Shalgunov et al. 2020).

### Oncology-theranostics

As is the case for any new RPs under development, theranostic RPs must undergo strict testing, which will provide important information on their biological behaviour, safety and suitability for clinical application (Kolenc Peitl et al. 2019). Some of the parameters evaluated during non-clinical testing are stability and affinity measurements, determination of targeting efficiency, biodistribution profile, metabolite identification and estimation of radiation and therapeutic efficacy as described in previous chapters. Concerning toxicology testing, theranostic RPs are required to undergo the same testing considerations as described in “Preclinical toxicology: from microdosing to a risk base approach” section.

There are two ways theranostics can be defined (1) using the same targeting molecule (e.g. an antibody) (Nakata et al. 2022), to which a radioisotope with a diagnostic or a therapeutic property is attached; (2) attaching a diagnostic radioisotope to a therapeutic agent (e.g.^89^Zr-trastuzumab) (Janjigian et al. 2013). In both approaches, it is assumed that the biological behaviour of both diagnostic and therapeutic vectors will be the same in vivo and the same targeting properties will be retained. The non-clinical evaluation testing, both in vitro and in vivo, has to prove these identical targeting properties of the diagnostic and therapeutic RP. This is, however, usually difficult to achieve in reality because of the differences in the processing of the radioisotope in question, its specific activity, and potential chemical and radionuclidic impurities. These, in turn, may influence the molar activity of the radiolabelled molecule. One needs to bear in mind that it has been demonstrated that the biodistribution of various RPs is mass-dependent (Luurtsema et al. 2021).

Therefore, in non-clinical studies the following factors need to be additionally considered:

- The production route of the radionuclide and its influence on the molar activity (and especially the effective specific activity) of the radiolabelled molecules with either the diagnostic or therapeutic radionuclide.
- The mass effect should be considered when assessing in vitro affinity of the radiolabelled molecules to the receptors overexpressed on cancer cells.
- Care should be taken when in vivo studies are performed, because mass adjustment may not always go together with the needed radioactivity to get adequate quality of images.

This is particularly important when the comparative evaluation of theranostic RPs is foreseen.

## Quality assurance and control measures

### Activity determination and dosimetry standardization

Standardisation and traceability are important components of quality assurance in activity assessment and dosimetry. There is no specific guidance published on this topic, so the first step in standardisation consists of guiding dosimetry reporting, similar to what has been done for diagnostic procedures in the clinical context (Lassmann et al. 2011). In general, quality control of activity measurement systems should be implemented, with regular checks and calicbrations of a frequency based on manufacturer guidance. This includes image-based (non-clinical PET/CT or SPECT/CT) and non-image-based (dose calibrators and gamma counters) activity determination devices.

Image-based activity determination should be performed considering a range of parameters that should be reported for traceability. These should cover the characteristics of imaging devices, acquisition and reconstruction procedures and should be detailed enough to allow repetition. Pharmacokinetic assessment and extrapolation should also be documented. Required information includes animal handling and preparation, physiological conditions during imaging, time sampling and time-activity curve fitting parameters. Estimates of the goodness of the fit should also be provided. Allometric extrapolation should be documented with references and sufficient data to allow reprocessing if needed. Finally, the dosimetry calculation scheme should be presented, clearly indicating that recommendations have been followed, mentioning the reference models, radiation weighting factors and the calculational procedure.

### Quality of the RP in non-clinical development

Acceptable quality control parameters are very important for diagnostic and therapeutic RPs. For clinical application it is mandatory to meet the guidelines for quality control recommended by regulatory authorities such as the European Pharmacopeia (EP), United States Pharmacopeia (USP), the WHO International Pharmacopoeia, or the Nuclear Regulatory Commission (NRC). Accordingly, specifications need to be defined and three separate batches are tested for radioactive, pharmaceutical, and chemical impurity parameters before the application of the RPs. Apart from the quality control of the RP product, the accuracy and constancy of the instruments used in the preparation and testing are also very important. There are no specific guidelines for quality assurance and quality control for the non-clinical use of the RPs. It has to be stressed that when moving to nonclinical in vivo studies, specifications need to be defined, which should be followed throughout the development process to ensure robustness of the data required for clinical translation. Table 5 provides an example of recommendations for quality control of a non-clinical preparation of a radiolabelled small molecule. However, such specifications will vary depending on the type of molecule, the mode of preparation and the radionuclide used. For example molar activity may be a parameter of particular importance in the non-clinical setting.

**Table 5:** Specifications for quality control of a novel RP: exemplified acceptance criteria and methods

Test	Acceptance criteria	Method reference
Appearance	Free from particle	Visual
pH	4.5–8	pH indicator paper
Radiochemical identity	Co-elution with unlabelled reference	LC
Radiochemical purity	> 95%	LC with radio detector
Radionuclide purity	> 99.9%	Gamma spectrometry
Chemical amount of UV-absorbing impurities a	The mass does not exceed 10 µg per injected dose	LC
Residual solvents DMSO	Not more than 5000 ppm	LC
Acetonitrile	Not more than 410 ppm	GC
Ethanol content	Not more than 10%	GC
Sterility	Sterile	Ph Eur

### Good laboratory practices (GLP)

According to the Directive 2004/10/EC, Good Laboratory Practice (GLP) is “a quality system concerned with the organizational process and the conditions under which non-clinical health and environmental safety studies are planned, performed, monitored, recorded, archived and reported” (Directive 2004). In Europe the OECD Advisory Document on GLP Data Integrity provides guidance for test facilities or test sites that conduct GLP studies and aims to promote a risk-based approach to data management (Practice and (GLP)-OECD [Internet]. 2021).

Non-clinical studies conducted during the development process of a novel pharmaceutical usually follow GLP regulations. During the non-clinical assessment process, a drug candidate is subjected to several evaluation steps, pharmacokinetics, ADME (absorption, distribution, metabolism and elimination) studies, safety pharmacology, and toxicity studies.

While it is generally expected that non-clinical safety studies are executed in compliance with GLP (Directive 2004), this may not be possible in the case of RPs, mainly due to radiation safety considerations (Guideline on the non-clinical requirements for radiopharmaceuticals [Internet]. 2018). Therefore, pharmacokinetic, biodistribution, and dosimetry studies are performed under non-GLP conditions. In contrast, non-clinical safety studies, in particular toxicity studies, are performed with the non-radioactive component according to GLP, in GLP-certified laboratories (Guideline on the non-clinical requirements for radiopharmaceuticals [Internet]. 2018). It needs to be emphasized here that the GLP certification of the laboratory performing toxicity studies is subject to national regulations and is a demanding process. Thus, in Europe these competences are offered almost exclusively by clinical research organizations (CRO) and at high cost. These organizations, however, are not prepared to carry out studies of radioactive products. Their offer is limited to the non- radioactive component of the investigational RP.

The initiative undertaken within this document aims to facilitate the conditions for non-clinical investigations of RPs to meet the regulatory constraints. However, in the current regulatory set up, the above does not apply to studies related to toxicity, even though the ‘microdose’ guideline applies in most cases. In the ‘investigational medicinal product dossier’ (IMPD) the toxicity data need to be supported by reports of the drug substance toxicity conducted in compliance with GLP.

In the US the FDA conducts careful inspections of facilities that perform nonclinical laboratory studies to determine compliance with Part 58 (Good Laboratory Practice for Nonclinical Laboratory Studies) of Title 21 of the Code of Federal Regulations. Nonclinical laboratory studies are experiments in which test articles are studied prospectively in test systems (animals, plants, microorganisms, or subparts thereof) under laboratory conditions to determine their safety (FDA. Affairs O of R. 2021). Previously, toxicology studies were performed in laboratories complying with good laboratory practice (GLP); however, recent U.S. changes through pre-IND meetings with the FDA have allowed these studies to be performed in other types of controlled laboratories, such as university comparative anatomy or veterinary medicine departments, which can further reduce non-clinical costs (Schwarz and Decristoforo 2019). Although this FDA ruling is not recognized by European or Canadian authorities (Schwarz et al. 2018), a recent position paper from the European Association of Nuclear Medicine (Koziorowski et al. 2017) and the EMA plans for specific RP guidance (Guideline on the non-clinical requirements for radiopharmaceuticals [Internet]. 2018) that will hopefully lead to more harmonized and rational approaches to preclinical safety data for new RPs.

## Conclusion

In this paper, we attempted to provide guidance on the translation of RPs from the preclinical stage into a clinical trial, particularly related to navigating the regulatory landscape. Today RPs are considered as drugs or Medicinal Products almost all over the world, independent of their diagnostic or therapeutic application. The information gained within the non-clinical development phase has to be submitted to drug-regulatory authorities with significant experience in conventional drugs, but many times limited expertise for the particular properties and requirements of RPs. Specific guidance is generally rare, also considering the wide range of applications that RPs can cover, involving a vast variety of different targets, different radionuclides requiring variable chemical approaches in the design, as well as the great range of potential clinical applications from oncology to neurology and from diagnosis to therapy. The recommendations given herein may help interpreting existing guidelines and, in combination with technical information, e.g. provided in a recent IAEA publication (Guidance for preclinical studies with radiopharmaceuticals. IAEA radioisotopes and radiopharmaceuticals series 2021), might help researchers, clinicians, and regulators to cope with requirements, ensuring that the application of a novel RP is safe and has a high potential to be successfully used in humans. Recent advances in drug development have led to even wider and more advanced applications of RPs involving nano-technologies or theranostics with novel radionuclide pairs. This also increases the need to educate the regulatory side. In a situation where a particular subclass of investigational RPs gains more widespread use, and adequate experience in terms of the relationship between non-clinical assessment data and human in vivo behaviour is gained, regulators should finally strive to issue guidance specific for RPs, in order to both educate the investigator community and to standardize the non-clinical evaluation methodologies applied. Such processes will also help strengthen RP development and allow a more rapid advance in this field with its high potential to benefit both clinical and research applications.

## Data Availability

Not applicable.

## Abbreviations

3Rs: Replace, reduce, refine
AC: Attenuation correction
ADME: Absorption, distribution, metabolism and elimination
BBB: Blood brain barrier
CRO: Clinical research organizations
CT: Computerized X-ray tomography
EMA: European Medicines Agency
EP: European pharmacopoeia
FDA: (US) Food and Drug Administration
FUS: Focused ultrasound
GLP: Good laboratory practice
GMP: Good manufacturing practices
IAEA: International Atomic Energy Agency
ICRP: International Commission on Radiological Protection
IMP: Investigational medicinal product
IMPD: Investigational medicinal product dossier
MA: Marketing authorization
MR: Magnetic resonance
NOAEL: No observed adverse effect level
NRC: Nuclear regulatory commission
OI: Optical imaging
PD: Pharmacodynamics
PK: Pharmacokinetics
RP: Radiopharmaceutical
SUV: Standardized uptake values
SUVR: SUV ratio
TAC: Time activity curve
TM: Technical meeting
TRT: Targeted radionuclide therapy
TTC: Threshold of toxicological concern
USP: United States pharmacopoeia
VOI: Volumes of interest
